# Regulation of TRIB3 mRNA and Protein in Breast Cancer

**DOI:** 10.1371/journal.pone.0049439

**Published:** 2012-11-20

**Authors:** Marloes Wennemers, Johan Bussink, Twan van den Beucken, Fred C. G. J. Sweep, Paul N. Span

**Affiliations:** 1 Department of Laboratory Medicine, Radboud University Nijmegen Medical Centre, Nijmegen, The Netherlands; 2 Department of Radiation Oncology, Radboud University Nijmegen Medical Centre, Nijmegen, The Netherlands; 3 Department of Radiation Oncology (Maastro Lab), Maastricht University, Maastricht, The Netherlands; University of Saarland Medical School, Germany

## Abstract

Tribbles homolog 3 (TRIB3) is a scaffold protein activated under hypoxic conditions and involved in several cell survival and proliferation pathways. Recently, we reported opposite associations of TRIB3 mRNA and protein with breast cancer prognosis. In this study, we investigated this discrepancy between TRIB3 mRNA and protein in human breast cancer. We provide several lines of evidence demonstrating that TRIB3 is a stabile protein which levels are not controlled by rapid protein breakdown. Interestingly, we were able to show that during anoxia TRIB3 mRNA translation was profoundly inhibited. Hypoxia induced micro RNA 24 was not responsible for the translational repression of TRIB3. Furthermore miRNA-24 expression levels in breast cancer patient specimens showed no correlation with TRIB3 mRNA or TRIB3 protein levels, or with prognosis. Thus, the expression of miRNA-24 does not explain the difference between mRNA and protein expression of TRIB3 in this cohort of breast cancer patients. In conclusion, TRIB3 protein is a stable protein which levels are predominantly regulated by translational control of TRIB3 mRNA transcript.

## Introduction

Tribbles homolog 3 (TRIB3) is a protein involved in multiple signaling pathways, including MAPK [1], TGF-beta [2], [3], NFκB [4], [5] and the PI3K pathway [6]. TRIB3 mRNA levels are upregulated by a variety of stresses, including hypoxia [7]. It is found that high TRIB3 mRNA expression is associated with a poor prognosis in both breast cancer and colon cancer [7], [8], [9]. Surprisingly, in the same group of breast cancer patients we found that TRIB3 protein expression had an opposite relation with prognosis [9]. High TRIB3 protein expression denotes a good prognosis. In oral tongue squamous cell carcinomas it was also found that TRIB3 protein expression was associated with a good prognosis [10].

These results spurred us to further investigate the control mechanisms responsible for TRIB3 protein expression. It is a general assumption that a gene is transcribed into mRNA, which is then directly translated into protein. The amount of transcribed mRNA thereby –at least partly- reflects the final amount of protein. Much of the mechanistic conclusions drawn from expression profiling studies in, for example, breast cancer are based on this premise. During the last decades, however, it has become clear that there are multiple ways to control protein levels, and that TRIB3 is a protein that might be tightly regulated on several levels.

Rzymski *et al.* showed that after anoxic exposure TRIB3 protein levels are increased at earlier time points than mRNA upregulation [4], suggesting rapid regulation of TRIB3 protein levels under hypoxia. Further, Ohoka *et al.* described that TRIB3 is an unstable protein that is ubiquitinated by cdh1 and thereby tagged for proteasomal degradation [11], a degradational process that plays an important role in protein level regulation. Hu-antigen R (HuR) also contributes to the increased expression of TRIB3 protein through increased translation and transcript stability under anoxia [4]. HuR is believed to bind to AU-rich elements in mRNAs and thereby it regulates both the stability and cytoplasmic/nuclear localization of mRNAs. One of the mechanisms potentially responsible for the discordance between TRIB3 mRNA and protein levels could be through expression of a class of small non-coding RNAs called microRNAs (miRNAs). miRNAs play an important role under both physiological and pathological conditions. Long pre-cursor miRNA called pri-miRNA are transcribed from the genome and are subsequently processed by the ribonucleases Drosha and Dicer into 21–24 nucleotide mature. In mammalian cells these mature miRNAs mostly hybridize imperfectly to the 3′UTR of target mRNAs and either cause mRNA instability or suppression of their translation. Due to the imperfect homology miRNAs can regulate multiple mRNA targets. It is estimated that all known miRNAs regulate up to 30% of the genes in the human genome [12], [13]. So far the only experimentally validated miRNA that targets TRIB3 is miRNA-24 (miRWalk [3], [14]). This miRNA is upregulated during hypoxia [3], [15], and is interestingly involved in similar signaling pathways as TRIB3 itself namely TGF-beta [3], [16], [17], [18] and MAPK signaling [19]. Furthermore it plays a role in cell cycle regulation [20], [21] and is frequently de-regulated in tumor cells [21]. Therefore, we hypothesized that miRNA-24 could be involved in TRIB3 mRNA translational regulation during hypoxia.

Here, we investigated the discrepancy between TRIB3 mRNA and TRIB3 protein levels. For this we analyzed protein degradation and mRNA translation rates of TRIB3 in breast cancer cell lines under normoxic and hypoxic conditions. Furthermore, we used a patient cohort to investigate if miRNA-24 could mediate translational inhibition of TRIB3 mRNA, and whether the level of miRNA-24 itself has prognostic value in breast cancer.

## Materials and Methods

### Ethics statement

As approved by the Radboud University Nijmegen Medical Centre Institutional Review Board and according to the “Code Proper Secondary Use of Human Tissue” developed by the Federation of Medical Societies (FMWV) in the Netherlands and Dutch national law, coded tumor tissues from anonymized patients were used. This study was performed according to REMARK guidelines [22].

### Patient samples

Frozen breast cancer tissue sections were available from in total 94 patients who had undergone resection of their primary tumor. The patient cohort is as described before with one failure due to missing miRNA 24 value [9]. Breast cancer patients were selected from a cohort treated between January 1991 and December 1996, that were not treated systemically, and had at least 5 years follow up or a recurrence before that. The selection criteria resulted in a patient cohort with 25% ER positive, 13% PR positive, 30% HER2-positive, and 44% triple negative patients.

### Breast cancer cell lines

Human breast cancer cells MDA-MB-231 and MCF-7 (ATCC, LGC Promochem, London, UK) were cultured for a limited number of passages in standard culture medium (DMEM with 10% dialyzed FCS, 2 mM L-glutamine, 20 mM HEPES, penicillin/streptomycin, and nonessential amino acids (all PAA Laboratories, Pasching, Austria)) at 37°C with 5% CO_2_, unless stated otherwise.

Knockdown of *TRIB3* was performed using siRNA transfection reagent SAINT-RED (Synvolux Therapeutics B.V., Groningen, the Netherlands). siRNA's MISSION® siRNA Universal Negative Control #1 (SIC001) and TRIB3 (SASI_Hs01_00197511) were acquired from Sigma-Aldrich (Sigma-Aldrich Chemie B.V., Zwijndrecht, the Netherlands). After transfection siRNA's were giving 24 hours to reduce TRIB3 mRNA levels and thereafter treated for 24 to 48 hours before harvesting of the cells.

For incubation under hypoxic conditions a hypoxic culture chamber was applied (H35 hypoxystation, Don Whitley Scientific, West Yorkshire, UK). Treatment of cells was performed with 1 or 10 µM of the proteasome inhibitor MG132 (Calbiochem, La Jolla, CA, USA), 50 µM chloroquine disphosphate salt (CQ, Sigma-Aldrich Chemie BV, Zwijndrecht, the Netherlands) and 100 µg/ml cycloheximide (CHX, Sigma-Aldrich Chemie BV). In the experiments where we combined treatment with hypoxia exposure compounds were added immediately before the cells were transferred into the hypoxic culture chamber.

### Western blot analysis

Cells were harvested in RIPA buffer (PBS (Klinipath, Duiven, the Netherlands), MQ (Sterile H_2_O, Versol®, Lyon, France), NP-40 (Sigma-Aldrich Chemie BV), Sodiumdesoxycholate (VWR International BV, Amsterdam, the Netherlands) and SDS) with phosphatase and protease inhibitors (Roche, Indianapolis, IN, USA). Samples were sonicated, centrifuged and supernatant was stored at −80°C. Protein quantification was performed using a Pierce® BCA Protein Assay Kit (Thermo Scientific, Etten-Leur, the Netherlands). Thirty microgram of protein was fractionated on 4–20% Criterion XT Bis-Tris gels (Bio-Rad Laboratories BV). After electrophoresis, samples were transferred to PVDF membranes (Millipore Immobilon, Millipore BV, Amsterdam, the Netherlands). Membranes were blocked with 5% NFDM (Blotting Grade Blocker Non-Fat Dry Milk, Bio-Rad Laboratories BV) and incubated with the appropriate antibodies (mouse anti-α-tubulin (1∶1000, Cell Signaling Technology, BIOKÉ, Leiden, the Netherlands) rabbit anti-β-catenin (1∶1000, Cell Signaling Technology) or chicken anti-TRIB3 peptide 3 (1∶500, characterized in [9])). Proteins were detected using chemiluminescent peroxidase substrate (Sigma-Aldrich Chemie BV) and visualized with a ChemiDoc XRS+imaging system (Bio-Rad Laboratories BV). Images and protein band intensities were acquired using Quantity One® 1-D Analysis Software (Bio-Rad Laboratories BV). Signal intensities were quantified using gel analyze tools from Image J [23] and corrected for α-tubulin intensities detected on the same blot.

## Polysomal Fractionation and Analysis

Polysomal fractionation and analysis was performed as described previously [24].

Cells were treated with 0.1 mg/ml cycloheximide (CHX) for 3 min at 37°C, washed twice with ice cold PBS/CHX and harvested by scraping in lysis buffer (1% Triton X-100, 0.3 M NaCl, 15 mM MgCl_2_, 15 mM Tris (pH 7.4), 0.1 mg/ml CHX, 100 units RNAse-In (Ambion)) at 4°C. Nuclei were removed and residual debris was removed by centrifugation. The lysate was layered on a10 ml continuous sucrose gradient (20–50% sucrose in 15 mM MgCl2, 15 mM Tris (pH 7.4), 0.3 M NaCl). After 90 min of centrifugation at 260,000 g at 4°C, the absorbance at 254 nm was measured continuously as a function of gradient depth in a BioRad Laboratories UV monitor. Translation efficiency was defined as the sum of the relative TRIB3 mRNA expression multiplied by the average number of ribosomes within each fraction divided by the total amount of TRIB3 mRNA.

### miRNA measurement

Total RNA from frozen tissue sections or cell cultures was isolated with the total RNA purification kit (Norgen Biotek Corp., Ontario, Canada). For the tissue sections an adjacent H&E stained section was used to determine the percentage of tumor cells in the tissue. Reverse transcriptase reaction was performed with NCode™ VILO™ miRNA cDNA Synthesis Kit (Invitrogen, Carlsbad, CA, USA) and miRNA expression was determined using Sybr Green Master Mix (Applied Biosystems, Nieuwerkerk a/d lJssel, the Netherlands) with miRNA-24 forward primer (gctcagttcagcaggaac) or RNU6 forward primer (cgcaaggatgacacgcaaattc, both Biolegio BV, Nijmegen, the Netherlands) and Universal qPCR primer (Invitrogen) on an CFX96 realtime PCR detection system (Bio-Rad Laboratories BV, Veenendaal, the Netherlands). All samples were normalized for levels of RNU6 expression.

### Statistical analyses

Statistical analyses were carried out using SPSS 16.0.5 software (SPSS Benelux BV, Gorinchem, the Netherlands). Normality of distribution of variables was tested using Kolmogorov-Smirnov testing. Differences were assessed with parametric unpaired t-tests and bonferroni's multiple comparison test or non-parametric Mann-Whitney U tests (for two groups) or with Kruskall-Wallis tests (for more than two groups) where appropriate. Non-parametric correlations were established using Spearman Rank correlation testing. Disease free survival (DFS) time (defined as the time from surgery until diagnosis of recurrent or metastatic disease) and overall survival (OS) time (defined as the time between date of surgery and death by any cause) were used as follow-up endpoints. Survival curves were generated using the method of Kaplan and Meier, after patients were categorized by miRNA-24 expression in either two or three equally sized groups, thus either at the p50, or at the p33 and p66. Equality of survival distributions was tested using log-rank testing, with Mantel-Cox test for trend when more than two groups were analyzed. Two-sided P-values below .05 were considered to be statistically significant.

## Results

### TRIB3 knockdown does not affect TRIB3 protein expression

**Figure 1:**
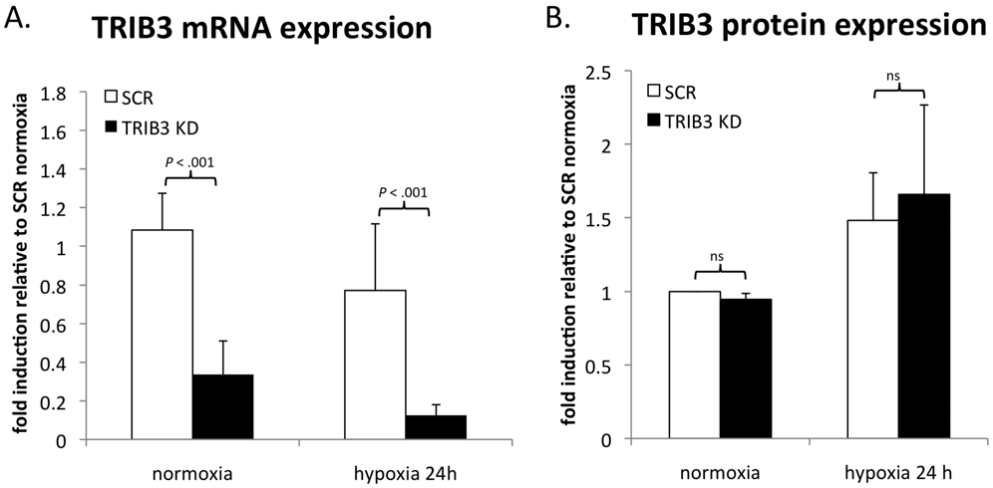
Effect of siRNA mediated knockdown of TRIB3 on mRNA and protein levels. A. Fold induction of TRIB3 mRNA in MDA-MB-231 cells during normoxia and after 24 hours of hypoxia (1% oxygen) treated with negative control siRNA (SCR) or siRNA mediated TRIB3 knockdown (TRIB3-KD) (n = 8). B. Fold induction of TRIB3 protein intensity in MDA-MB-231 cells during normoxia or after 24 hours of hypoxia (1% oxygen) treated with negative control siRNA (SCR) or siRNA mediated TRIB3 knockdown (TRIB3-KD) (n = 3). Protein intensities were controlled for α-tubulin intensities on same blot. Fold induction are averages (+ SD) relative to levels in cells treated with control siRNA (SCR) during normoxia.

During our studies to investigate the biological relevance of TRIB3 for the adaptation to hypoxic conditions we developed a TRIB3 knockdown model. This siRNA approach was successful in decreasing TRIB3 mRNA levels compared to cells treated with negative control siRNA (SCR) during normoxia and hypoxia (*P* = <.001) (Figure 1A). Even though the mRNA levels were profoundly reduced by the siRNA mediated knockdown the protein levels did not diminish in these cells neither under normoxic nor hypoxic conditions (*P* = .80) (Figure 1B). This data suggests that TRIB3 is a very stable protein.

### TRIB3 protein degradation

**Figure 2:**
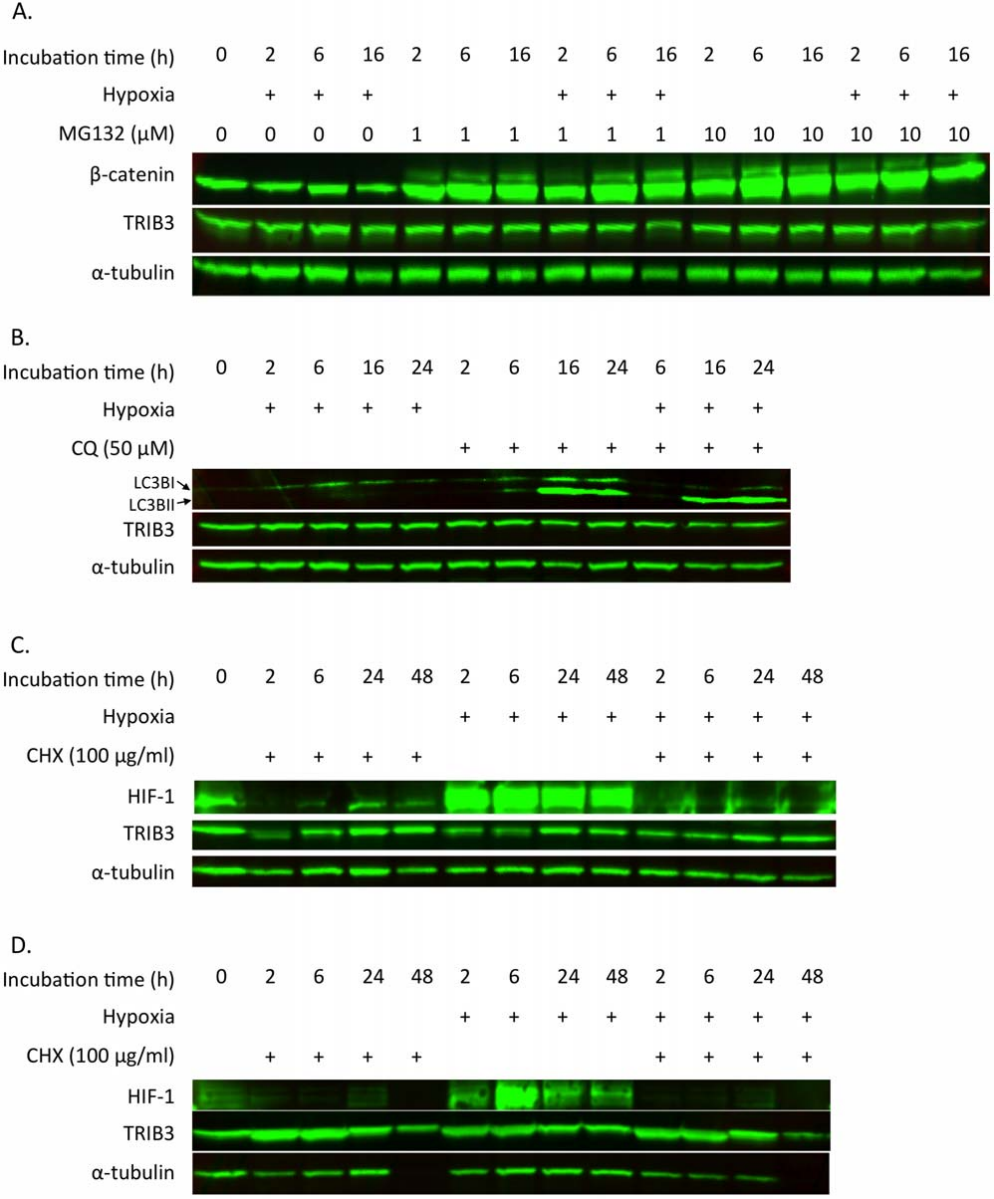
Effect of proteasome inhibition on TRIB3 protein levels. A. Western blot of MDA-MB-231 cells treated with MG132 and exposed to hypoxia (0.1% oxygen) immunostained for β-catenin (positive MG132 control), TRIB3 and α-tubulin (loading control). B. MDA-MB-231 cells treated with hydroxychloroquine (CQ) and exposed to hypoxia (1% oxygen) immunostained for LC3b (positive autophagy inhibition marker), TRIB3 and α-tubulin (loading control). C. MDA-MB-231 cells treated with cycloheximide (CHX) and exposed to hypoxia (1% oxygen) immunostained for HIF-1 (positive translation inhibition control), TRIB3 and α-tubulin (loading control). D. MCF-7 cells treated with cycloheximide (CHX) and exposed to hypoxia (1% oxygen) immunostained for HIF-1 (positive translation inhibition control), TRIB3 and α-tubulin (loading control).

To determine the rate of protein degradation through the proteasome we treated breast cancer cells with the proteasome inhibitor MG132. MDA-MB-231 cells were exposed to 0, 2, 6, or 16 hours of hypoxia (0.1% oxygen) and treated with MG132. In the cell-lysates from these cells we determined protein concentrations of β-catenin, TRIB3 and α-tubulin using western blotting (Figure 2A). β-catenin protein expression was used as a positive control to confirmed the effect of MG132 on proteasomal degradation. This protein was clearly more abundant in all samples treated with MG132, confirming the inhibition of proteosomal degradation in these samples. However, TRIB3 protein levels did not change after treatment with MG132 nor after exposure to hypoxia, indicating that TRIB3 is not degraded by the proteasome. Thus, proteosomal degradation is not involved in the apparent strict regulation of TRIB3 protein levels in breast cancer cells.

Next we assessed whether TRIB3 protein levels might be affected through the autophagic pathway. Chloroquine (CQ) was used to block autophagy allowing us to measure the autophagic flux in MDA-MB-231 cells that were culture under normoxic or hypoxic (1% oxygen) conditions for 2, 6, 16 or 24 hours. Treatment with CQ under normal conditions resulted in an increased accumulation of LC3bI and II consisted with a blocked autophagic pathway (Figure 2B). Under hypoxic conditions the LC3BII form accumulates indicating a high flux through the autophagic pathway and thus more active autophagy. Blocking autophagy however did not lead to an increase in TRIB3 protein expression. This data rules out autophagy as a potential regulator of TRIB3 protein levels.

### The protein stability of TRIB3

Since in our hands neither inhibiting the proteasome nor inhibiting the autophagy pathway seems to effect TRIB3 protein expression in MDA-MB-231 cells the stability of the protein was determined using a translational inhibitor cycloheximide (CHX). To test whether the TRIB3 breakdown rate is cell line dependent we used MDA-MB-231 as well as MCF-7 cells. Cells were exposed to CHX during normoxic or hypoxic (1% oxygen) conditions for 2, 6, 24 or 48 hours. HIF-1 expression was used as a positive control for the translational inhibition. During normoxic conditions HIF-1 protein was barely detectable using western blotting in both cell lines (Figure 2 C and D), consisted with the extremely short half-life of HIF-1 under aerobic conditions. As expected hypoxic conditions results in remarkably higher HIF-1 protein levels due to the inhibition of oxygen dependent degradation of HIF-1. Translational inhibition reduced HIF-1 levels during normoxia and this effect was more obvious during hypoxia. In the MDA-MB-231 cells TRIB3 protein levels did not change as a result of translational inhibition. For MCF-7 cells an increase is seen in TRIB3 levels both during normoxia and hypoxia when treated with CHX for 2 and 6 hours. A decrease in TRIB3 levels is seen in the MCF-7 cells treated with CHX for 48 hours, however this decrease is also observed for α-tubulin. CHX treatment for 48 hours was toxic to the cells and the reduction in the number of cells used for protein extraction led to a lower protein amount loaded on the gel. These results show that TRIB3 is a relative stable protein with half lives comparable to α-tubulin under both normoxia and hypoxia.

### TRIB3 mRNA translation is reduced by anoxic exposure

**Figure 3:**
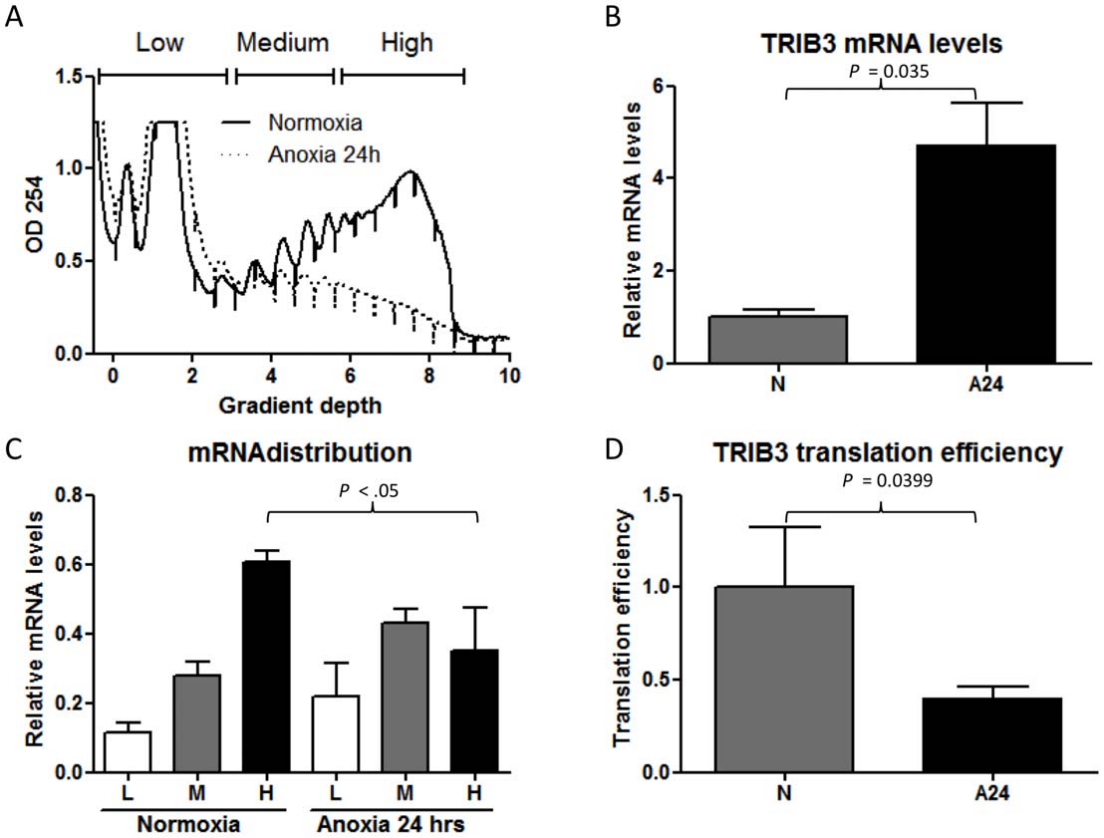
TRIB3 mRNA translation during anoxia. A. Polysome profiles of MCF7 cells grown under aerobic of anoxic conditions during 24 hours. B. TRIB3 mRNA levels determined by Q-PCR in the total RNA fraction. C. TRIB3 mRNA distribution within the polysome. D. Change in translation efficiency of the TRIB3 mRNA after exposure to anoxia for 24 hrs. Experiments were performed in triplicates.

To further investigate the discrepancy between TRIB3 overall mRNA and protein levels we measured TRIB3 mRNA translation upon anoxia. RNA isolated from MCF7 cells was separated through sucrose gradients based on the number of ribosomes associated with the RNA. Exposure to anoxic conditions for 24 hrs caused an inhibition of general translation (Figure 3A). TRIB3 mRNA levels were ∼5-fold up regulated in MCF7 cells upon anoxia (*P = .035*, Figure 3B). Nevertheless anoxia caused a change in the distribution of the TRIB3 mRNA within the polysome. More TRIB3 mRNA was detected within the fractions containing less ribosomes (fractions L+M) compared to aerobic conditions where most of the TRIB3 mRNA is associated with many ribosomes (H fraction) (Figure 3C). As the number of polysomes on the RNA reflects the extent of translation into protein, these results indicate that anoxia inhibits the translation of TRIB3 mRNA into protein. Finally, we calculated the translation efficiency for TRIB3 as described in the material and methods. Consistent with the tendency of TRIB3 mRNA to be associated with less ribosomes, the translation efficiency is profoundly reduced under anoxic conditions (*P* = .0399, Figure 3D). This data suggest that translational control of TRIB3 mRNA can explain discrepancies between TRIB3 mRNA abundance and protein levels, especially under oxygen deprived conditions.

### miRNA-24 expression in breast cancer cells during hypoxia

**Figure 4:**
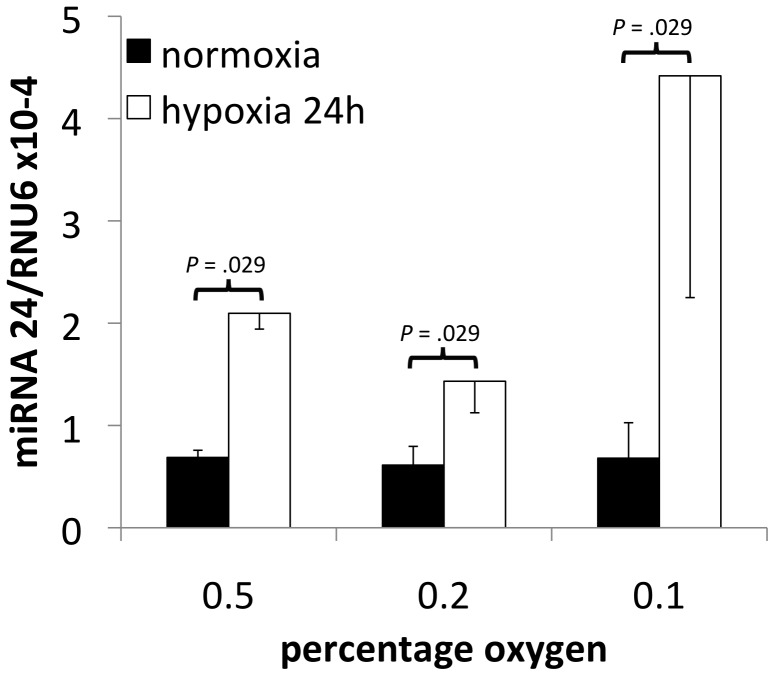
miRNA-24 expression after hypoxia. Expression levels (average +/− SD, n = 4) measured by RT-qPCR of miRNA-24 controlled for RNU6 expression in MDA-MB-231 cells exposed to 0.5%, 0.2% or 0.1% oxygen during 24 hours.

Repression of TRIB3 mRNA translation could potentially be mediated through hypoxia induced miRNAs. Analysis of the 3′UTR of TRIB3 using the prediction algorithm TargetScan revealed miRNA24 as a potential candidate [13], [25]. First we determined whether hypoxia indeed upregulates miRNA-24 levels in breast cancer cells during hypoxia. For this we quantified miRNA-24 expression in MDA-MB-231 breast cancer cells exposed for 24 hours to 0.5%, 0.2%, or 0.1% oxygen. Hypoxia indeed caused an induction in miRNA-24 expression ranging from 2.4 to 6.5 fold (*P* = 0.029). The strongest induction was observed in the cells exposed to the lowest oxygen concentration (Figure 4). Thus, miRNA-24 is a potential candidate marker that could explain the opposite association of TRIB3 mRNA and protein with breast cancer prognosis.

### miRNA-24 expression in breast cancer patients

Next, we assessed whether an inverse correlation exists between miRNA-24 and TRIB3 mRNA or protein levels in breast cancer patients. miRNA-24 expression was detectable in all 94 available breast tumor samples. The miRNA-24 levels were not log-normally distributed and therefore non-parametric statistical tests were used. TRIB3 mRNA and protein expression were determined and scored as described before [9]. There was no correlation between miRNA-24 levels and mRNA (r_s_ = −.091, *P* = .383) or protein (r_s_ = .009, *P* = .932) levels of TRIB3 (Table 1). Next, patients were divided into 6 groups based on low or high TRIB3 mRNA expression and negative, weak or strong TRIB3 protein expression. There was no difference in miRNA-24 expression between these groups (*P* = .7, Table 2), indicating that miRNA-24 could not explain the discrepancy in TRIB3 mRNA and protein levels in these patients.

**Table 1:** Correlation between miRNA-24 expression and TRIB3 mRNA and protein expression.

		TRIB3 mRNA	TRIB3 protein
miRNA-24	Correlation Coefficient^a^	−.091	.009
	*P*	.383	.932
	n	94	94
TRIB3 protein	Correlation Coefficient^a^	−.043	
	*P*	.683	
	N	94	

a Spearman's rho.

**Table 2:** miRNA 24 expression levels in groups with accordance and discordance between TRIB3 mRNA and protein.

	TRIB3 protein score	negative	weak	Strong
TRIB3 mRNA				
<median	n	14	27	6
	median miRNA24/RNU6 (*10^−3^)	0.8	0.89	0.9
	interquartile range miRNA24/RNU6 (*10^−3^)	1.49	1.43	0.99
>median	n	17	24	6
	median miRNA24/RNU6 (*10^−3^)	0.88	0.6	1.15
	interquartile range miRNA24/RNU6 (*10^−3^)	1.34	1.16	0.9

**Figure 5:**
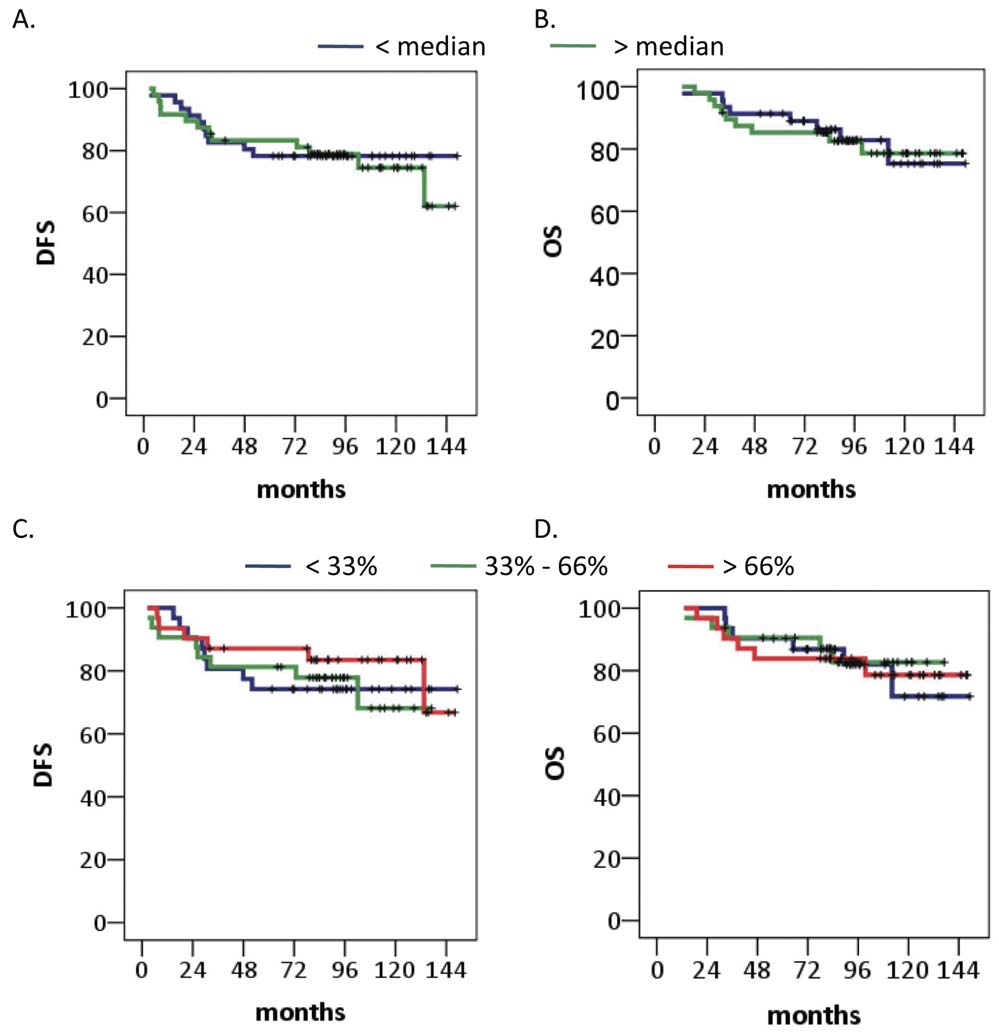
Breast cancer patient survival based on miRNA-24 expression levels. A. Disease free survival in patients stratified according to miRNA-24 expression below or above median. B. Overall survival in patients stratified according to miRNA-24 expression below or above median (0.9×10^−3^). C. Disease free survival in patients stratified according to tertiles miRNA-24 expression below 0.5×10^−3^, above 1.2×10^−3^ or in between. D. Overall survival in patients stratified according to tertiles miRNA-24 expression below 0.5×10^−3^, above 1.2×10^−3^ or in between.

Finally, we determined whether miRNA-24 expression on its own has any clinical value. miRNA-24 expression levels exhibited no association with any clinicopathological characteristic tested besides histological grade (grade I/II vs. III, 0.610 vs 0.910 median miRNA expression *10^−3^, *P* = .02) (Table 3). Furthermore, miRNA-24 did not show a relation with DFS or OS when the patient group was split according to expression levels into 2 groups at the median (*P* = .83 and *P* = .93) or into 3 groups at 33% and 66% (*P* = .73 and *P* = .96) (Figure 5).

**Table 3:** Associations of *miRNA-24* expression levels with clinicopathological characteristics.

Variable	N = 94^a^	Median (*10^−3^ miRNA-24/RNU6)	Interquartile range (*10^−3^ miRNA-24/RNU6)	*P* b
Age (years)				
<50	28	0.755	0.75	0.38
≥50	64	0.915	1.40	
Menopausal status				
Premenopausal	26	0.795	0.87	0.58
Postmenopausal	68	0.890	1.41	
Tumor type				
Ductal	75	0.840	0.99	0.87
Lobular	8	1.060	1.84	
Other (mixed/unknown)	11	0.940	1.19	
Tumor size				
pT1	49	0.870	0.98	1.00
pT2	40	0.840	1.41	
pT3/4	4	0.875	1.18	
Histological grade				
I/II	22	0.610	0.75	0.02
III	53	0.910	1.32	
Estrogen Receptor				
negative	65	0.880	1.02	0.69
positive	27	0.710	1.16	
Progestrone Receptor				
negative	77	0.890	1.38	0.40
positive	15	0.670	0.67	
Human Epidermal Growth Factor Receptor 2				
negative	67	0.830	1.36	0.90
positive	25	0.890	0.92	

a Due to missing values, numbers do not always add up to 94.

b *P* tested with Mann-Whitney U test or Kruskal-Wallis Test where appropriate.

## Discussion

Recently it has been described that patients with tumors that express more TRIB3 mRNA exhibit a poor prognosis [7], [8], [9], whereas in patients tumors with high levels of TRIB3 protein as established by immunohistochemistry were associated with a good prognosis [9], [10]. TRIB3 protein expression is most likely related to function and it is, therefore, that TRIB3 is probably functionally associated with a good prognosis. Indeed, previously TRIB3 has been found to be involved in ER stress induced cell death [26], [27], [28]. The important role for TRIB3 in cell survival under hypoxic conditions was indicated by the observation that after knockdown of TRIB3 cell survival was increased under hypoxia [9]. Thus, the relation of TRIB3 protein with a good prognosis in breast cancer patients could be due to its role in hypoxia-induced cell death. Tumors that are more hypoxia sensitive are less aggressive and respond better to for instance radiotherapy [29], [30]. On the other hand, the finding that high TRIB3 mRNA is associated with a poor prognosis suggests that any mechanism that is involved in the induction of TRIB3 mRNA is also involved in tumor progression and/or treatment resistance. Data supports that the CHOP/ATF4 arm of the unfolded protein response (UPR) which is responsible for TRIB3 upregulation during hypoxia [7] protects tumor cells during hypoxia [31].

The discrepancies between TRIB3 mRNA and protein and their opposing relation with breast cancer prognosis spurred us to investigate the regulatory mechanisms involved in TRIB3 translation. siRNA mediated knockdown of TRIB3 mRNA had no effect on TRIB3 protein levels in breast cancer cells. Further, inhibition of proteasomal degradation or autophagy had no appreciable effect on TRIB3 protein levels during normoxia or hypoxia and inhibition of translation indicated TRIB3 to be a stable protein. The upregulated of TRIB3 levels after inhibition of translation that was observed could be due to an effect on a protein that is involved in TRIB3 protein regulation. We could confirm that the only so far known TRIB3 specific micro RNA (miRNA-24) was upregulated during hypoxia in breast cancer cells. However, miRNA-24 levels did not correlate with either mRNA or protein expression of TRIB3 in breast cancer patients. While miRNA-24 has previously been described as a cell type-specific oncogene and tumor suppressor [21], [32], [33], [34] we did not find any prognostic value for miRNA-24. Martin et al. suggest that the passenger strand miR-24-2*, which does not bind TRIB3, might be involved in the opposing oncogenic and tumor suppressive roles of miRNA-24 [35].

TRIB3 mRNA is induced by hypoxia and ER stress via the PERK/ATF4/CHOP arm of the unfolded protein response (UPR) [7], [26], [36]. In addition, the NFκB pathway regulates TRIB3 expression after anoxia and Hu-antigen R (HuR) stabilizes the mRNA of TRIB3 [4]. Other pathways in which TRIB3 is involved are the MAPK [1], TGF-beta [2], [3] and PI3K pathway [6], [37], [38], [39]. Interestingly, TRIB3 reportedly constitutes a feedback loop with almost all of these pathways [1], [2], [4], [6], [27], [40], [41]. The combination of all these pathways and multiple feedback loops contributes to a tight regulatory control of TRIB3 emphasizing the importance of TRIB3 protein function. TRIB3 is involved in regulating multiple pathways in cell survival and could thereby affect cell survival and cancer prognosis. Understanding TRIB3 protein regulation mechanisms in more detail could provide valuable information for means to regulate cancer cell survival.

Rzymski *et al.* explored regulation mechanisms that could contribute to the control of TRIB3 [4]. They determined that the anoxia-induced upregulation of TRIB3 protein is faster than, and therefore probably not dependent on, TRIB3 mRNA upregulation [4]. Their focus was mainly on the regulation of mRNA levels rather than on protein levels. Here we studied mechanisms that effect protein regulation, as we found that attenuating TRIB3 mRNA levels using siRNA had no effect on TRIB3 protein levels. TRIB3 protein has been described to be tightly regulated by proteasomal degradation via ubiquitination by cdh1 [11], [42]. An unstable protein is for its stimulation more dependent on the degradation rate than on the mRNA expression as protein levels could increase quickly when protein degradation is diminished without increasing levels of mRNA. This could explain the more rapid increase of TRIB3 protein compared to mRNA under anoxic conditions [4]. We could not find any effect of inhibiting proteasomal degradation on the TRIB3 protein levels in breast cancer cells both during normoxia and hypoxia. The UPR has also been described to play a role in the activation of the autophagy pathway, which can also degrade proteins. Inhibiting autophagy did also not affect TRIB3 protein levels. Therefore, we wondered whether TRIB3 protein was in breast cancer cells as unstable as suggested before [4]. In fact, inhibiting the translation showed that TRIB3 protein is stable throughout 48 hours under both normoxic and hypoxic conditions. Being a stable protein both under normoxic and hypoxic conditions the discrepancies between mRNA and protein are more likely to arise in the translational step between them. Our observation that TRIB3 mRNA translation is reduced under anoxic conditions even though mRNA levels are increased further strengthens this hypothesis. One possible explanation could be that activation of the UPR leads to an upregulation of TRIB3 mRNA but also results in general translational inhibition [24]. However, a negative feedback loop via GADD34 leads to the dephoshorylation of eIF2α and thereby might promote recovery from this translational inhibition [43]. miRNAs are also capable of inhibiting translation of the mRNA transcript. Thus, TRIB3 specific miRNAs such as miRNA-24 (miRWalk, [3], [14]) might attenuate TRIB3 protein levels even in the presence of high mRNA levels. We found that miRNA-24 is hypoxia regulated in breast cancer cells, which is in line with what has been reported for other cell types [3], [15]. The exact oxygen tension might be of relevance for TRIB3 protein regulation. TRIB3 mRNA levels increase at both 0.1 and 1% oxygen, whereas TRIB3 protein only increases at 1% oxygen. As we found, this is not caused by increased degradation via the proteasome at 0.1% oxygen. Possibly, the induction of miRNA-24 at 0.1% oxygen precludes TRIB3 mRNA from being translated to TRIB3 protein. However, we did not find a relation between miRNA-24 and TRIB3 protein or mRNA. Although miRNA-24 was the miRNA with the lowest p-value (<.001) of the 9 predicted miRNA's for TRIB3 (miRWalk, [14]), it could well be that other miRNAs are responsible for the discrepancies found.

In conclusion, we showed that TRIB3 is a stable protein and that translational control is most likely responsible for the discrepancies between TRIB3 mRNA and protein. Further research is needed to understand the mechanisms through which TRIB3 is translationally regulated in more detail.
